# Early wound healing and refractive response of different pocket configurations following presbyopic inlay implantation

**DOI:** 10.1371/journal.pone.0172014

**Published:** 2017-02-24

**Authors:** Aris Konstantopoulos, Yu-Chi Liu, Ericia Pei Wen Teo, Nyein Chan Lwin, Gary Hin Fai Yam, Jodhbir S. Mehta

**Affiliations:** 1 Singapore Eye Research Institute, Singapore, Singapore; 2 Singapore National Eye Centre, Singapore, Singapore; 3 University Hospital Southampton NHS Foundation Trust, Southampton, United Kingdom; 4 Department of Clinical Sciences, Duke-NUS Graduate Medical School, Singapore, Singapore; 5 Nanyang Technological University, Singapore, Singapore; Chang Gung University, TAIWAN

## Abstract

**Background:**

Presbyopic inlays have mostly been implanted under a corneal flap. Implantation in a pocket has advantages including less postoperative dry eye and neurotrophic effect, and better biomechanical corneal stability. This study investigated the effect of different pocket and flocket dimensions on corneal stability and refractive power after Raindrop™ implantation, and the associated wound healing response.

**Methodology:**

Ten New Zealand White rabbits had bilateral pocket Raindrop™ implantation. Eyes were allocated to 4 groups: pockets with 4mm, 6mm, and 8mm diameters, and 8mm flocket. They were examined pre-operatively, at day 1, weeks 1, 2, 3 and 4 post-surgery with anterior segment optical coherence tomography, corneal topography and in-vivo confocal microscopy. After euthanasia (week 4), CD11b, heat shock protein (HSP) 47 and fibronectin corneal immunohistochemistry was performed.

**Results:**

Corneal thickness (mean±SD) increased from 360.0±16.2μm pre-operatively to 383.9±32.5, 409.4±79.3, 393.6±35.2, 396.4±50.7 and 405±20.3μm on day 1, weeks 1,2,3 and 4 respectively (p<0.008, all time-points). Corneal refractive power increased by 11.1±5.5, 7.5±2.5, 7.5±3.1, 7.0±3.6 and 6.3±2.9D (p<0.001). Corneal astigmatism increased from 1.1±0.3D to 2.3±1.6, 1.7±0.7, 1.8±1.0, 1.6±0.9 and 1.6±0.9D respectively (p = 0.033). CT, refractive power change and astigmatism were not different between groups. The 8mm pocket and 8mm flocket groups had the least stromal keratocyte reflectivity. CD11b, fibronectin or HSP47 weren’t detected.

**Conclusions:**

Anatomical and refractive stability was achieved by 1 week; the outcomes were not affected by pocket or flocket configuration. No scarring or inflammation was identified. The 8mm pocket and flocket showed the least keratocyte activation, suggesting they might be the preferred configuration.

## Introduction

Presbyopia, a physiological change in near vision due to loss of accommodative power of the crystalline lens, affects individuals older than 40 years.[1] Population ageing is a major demographic trend worldwide and it is expected that the global population with presbyopia will increase from 1.2 billion in 2010 to 1.8 billion by 2050.[2] In addition, the widespread adoption of personal computers and smartphones, combined with the good health of the ‘baby boomers’, has imposed addition pressure on ophthalmologists to facilitate near and intermediate vision activities.

The vast majority of the population compensates for presbyopia with the use of spectacles with convex lenses. This has the disadvantage that objects are in sharp focus only at one predetermined distance and spectacles may need to be changed to a second pair for intermediate vision or removed for distance. Bifocal or varifocal lenses, however, can overcome this limitation, making spectacles an inexpensive and safe option. Many patients, though, are reluctant to start wearing spectacles for reasons such as inconvenience, cosmesis and social perception.

Spectacle independent compensation for presbyopia can be achieved with monovision, multifocal contact lenses, multifocal intraocular lenses, conductive keratoplasty and corneal laser surgery.[3] All have potential advantages and disadvantages, but a common disadvantage is a reduction in the overall quality of vision. Patients with monovision develop reduced stereo-acuity and binocular contrast sensitivity.[4,5] Multifocal intraocular lenses may result in dysphotopsia, such as night glare and haloes, and reduced contrast sensitivity.[6–8] Corneal laser presbyopic correction can have similar side effects, including haloes and glare and loss of distance visual acuity,[9–11] and also is irreversible.

Corneal inlays are an emerging new option for the surgical compensation of presbyopia. The safety and precision of femtosecond laser technology, demonstrated by the greater predictability in LASIK flap thickness with femtosecond laser compared to microkeratome flaps,[12] has also been a factor in the adoption of this new technology. An advantage of all inlays is that they can be removed or replaced and there is potential for enhancement should the refractive requirements of the patient change.[3]

Inlays can be typically placed under a flap,[3] or in a stromal pocket. The vast majority of inlays, to date, have been implanted under a corneal flap. The ability to create accurate corneal pockets, has become possible with the progress in femtosecond laser technology. However, some femtosecond laser platforms are unable to construct a conventional pocket with a lamellar cut and tunnel, and hence are only able to construct a flocket, a hybrid of a flap and pocket, as an alternative to a pocket.[13] A flocket consists of a conventional flap creation, but the hinge width is extended to 330 degrees, leaving only a small rim cut. A pocket or a flocket may have advantages over a flap, including less flap related complications,[14] e.g. less dry eye and neurotrophic ocular surface effects,[15–17] but implantation and inlay centration may be more difficult and simultaneous refractive adjustment may not be possible.

Currently, there are four CE (Conformité Européene) approved corneal inlays.[3] The Raindrop™ (ReVision Optics, Lake Forest, CA), a newer corneal inlay that is increasingly being used, has shown promising results for the compensation of presbyopia when implanted under a corneal flap and has recently been FDA approved.[18–21] It is approximately 10 μm thick at the periphery and 32 μm at the center, measuring 2 mm in diameter.[3]

All clinical studies to date have used a flap.[18–21] As implantation of the Raindrop inlay in a pocket or flocket has not been presented in the peer reviewed literature, we investigated the effect of different corneal pocket or flocket dimensions and configurations on the refractive outcome and corneal stability after Raindrop™ inlay implantation in a rabbit model. We also investigated the associated wound healing response.

## Materials and methods

### Animals and study design

Eleven New Zealand White rabbits (3–4 kg body weight), procured from the National University of Singapore, were housed at the animal holding unit of Singapore Eye Research Institute. Trained staff provided food and water regularly. The animals were transported from the animal housing room to the procedure room in individual rabbit transport cages; sedated rabbits were carried by hand. The cages were fully enclosed and had small openings, sufficient for the rabbits to breathe comfortably.

Ten rabbits had bilateral Raindrop™ inlay implantation; the inlay is FDA approved and does not cause visual disability that would disrupt the animals’ daily activities. The rabbits were anaesthetised with xylazine hydrochloride (5 mg/kg intramuscularly; Troy Laboratories, Smithfield, Australia) and ketamine hydrochloride (50 mg/kg intramuscularly; Parnell Laboratories, Alexandria, Australia) during surgery and examinations. Immediately following anaesthesia, frequent and careful observation of the rabbits was carried out. The animals were kept warm and returned to their cage when able to walk. Breathing and gum colour were monitored. Humane endpoint criteria were set; at any given time during the study, animals that suffered from severe or chronic pain and distress that could not be relieved with therapeutic intervention would be painlessly euthanized. The rabbits were euthanized at the end of the study under anaesthesia by overdose intracardiac injection of sodium pentobarbitone (Jurox, Rutherford, Australia). One rabbit was used as a non-surgical control. All animals were treated according to the guidelines of the Association for Research in Vision and Ophthalmology's Statement for the Use of Animals in Ophthalmic and Vision Research. The protocol was approved by the Institutional Animal Care and Use Committee of SingHealth (ref 2014/SHS/ 971).

The eyes of the 10 rabbits were allocated to 4 surgical groups, each group consisting of 5 eyes. The groups were: pocket with a diameter of 4 mm, pocket of 6 mm, pocket of 8 mm and an 8mm diameter flocket, as detailed below. The rabbits were examined under anesthesia before surgery, at day 1, and weeks 1, 2, 3 and 4 following surgery. They were euthanized after week 4 examinations.

### Surgery

#### Corneal stromal pocket procedure

**Fig 1:**
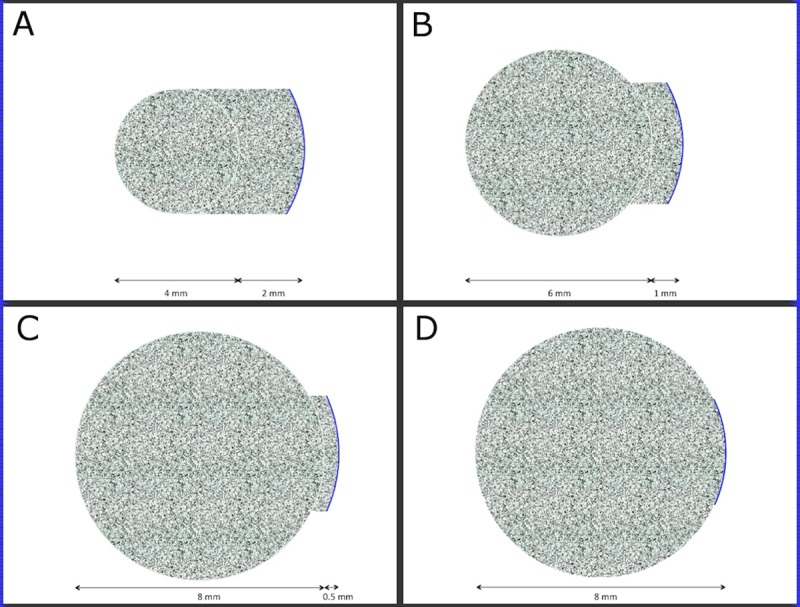
Schematic diagrams showing the design and dimensions of the femtosecond laser pockets and flocket. Figures A, B and C illustrate the 4 mm, 6 mm and 8 mm pockets respectively, figure D the 8 mm flocket. The blue line represents the access incision.

Pocket creation was performed using a Ziemer femtosecond laser system (Ziemer FEMTO LDV Z6, Ziemer Ophthalmic Systems AG, Port, Switzerland). The handheld laser delivery system was placed onto the cornea. After contact was made between the 9.5 mm suction ring and the corneal surface, suction was applied to the interface and the procedure performed. The laser parameters were: depth 170 μm, pulse energy 10 nJ, pulse frequency >5 MHz, spot size 1 μm with overlapping spots.[13] A planar lamellar laser incision was made with diameters of 4, 6 and 8 mm depending on the group. This was followed by creation of an access tunnel of 4 mm width; the length was 2, 1 and 0.5 mm, respectively, with an entry side cut angle of 90 degrees (Fig 1). Total energy per pocket was 0.048, 0.108 and 0.192 J respectively. The pocket was opened through the 4 mm entry incision with a Seibel spatula (Rhein Medical Inc., Petersburg, FL) and the inlay inserted as described below.

#### Flocket procedure

The flocket procedure was performed with the Visumax Femtosecond Laser (VisuMax, Carl Zeiss Meditec, Jena, Germany). A small curved interface cone was used for application of suction. The laser parameters were: anterior cap depth 170 μm, 200-nJ power, spot distance and tracking spacing of 3 μm/3 μm for lamellar and 2 μm/2 μm for flocket side cuts. Once suction was applied, a planar lamellar laser incision with an 8 mm diameter was made, followed by an arcuate access incision 30 degrees of arc, with a 90 degrees cut angle and at a diameter of 8 mm; total flocket energy was 1.422 J (Fig 1). The flocket was opened through the incision with a Seibel spatula (Rhein Medical Inc., Petersburg, FL) and the inlay inserted as described below.

#### Inlay implantation

**Fig 2:**
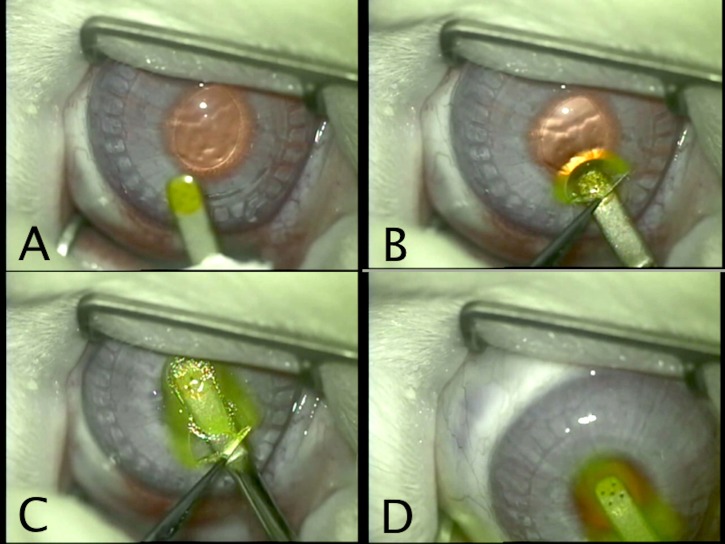
Implantation of the Raindrop inlay. A. Fluorescein stained inlay on the introducer. B. Introduction of the inlay through the access incision. C. Advancement of the inlay in the flocket. D. Release of the inlay from the introducer.

The Raindrop™ inlay was inserted into the stromal pocket or flocket through the access tunnel or incision using an inlay-specific introducer, provided by ReVision Optics. Placement was aimed for the centre of the pupil that was constricted preoperatively with pilocarpine 2% drops. In order to aid visualization of the inlay during loading and insertion, the inlay was stained with fluorescein 2% drops (Fig 2). After surgery, 0.3% Tobramycin drops were administrated four times daily for 1 week and 1% prednisolone acetate eye drops four times daily for 4 weeks.

### Investigations

The following investigations were performed under anaesthesia before and after surgery: slit lamp biomicroscopy photography (Righton, Tokyo, Japan), anterior segment optical coherence tomography (RTVue; Optovue, Inc, Fremont, CA), anterior corneal surface topography (ATLAS, Carl Zeiss Meditec) and in vivo confocal microscopy (Heidelberg Retina Tomograph—Rostock Cornea Module). A lid speculum was used to keep the rabbit eye open during imaging. The cornea was kept wet regularly with balanced salt solution to prevent the ocular surface from drying. An experienced technician held the anaesthetized rabbit in position so that the examined eye was perpendicular to the imaging device. After euthanasia, the rabbit corneas were examined with immunofluorescence for CD11b, heat shock protein (HSP) 47 and fibronectin.

#### Slit lamp biomicroscopy photography

Slit-lamp photos we taken before surgery, on day 1 and weeks 1, 2, 3 and 4 following surgery. They were examined for the presence of conjunctival injection, corneal haze, corneal scar and corneal infiltration.

#### Anterior segment optical coherence tomography

Anterior segment optical coherence tomography (AS-OCT) scans of the cornea were carried out through the center of the corneal inlay at the 180° axis before surgery, on day 1 and weeks 1, 2, 3 and 4 following surgery. The images were examined for stromal necrosis, by investigating for the presence of focal corneal thinning above and below the inlay. Corneal thickness (CT) was measured in the center of the inlay and analyzed serially in order to assess when corneal stability was achieved.

#### Anterior corneal surface topography

ATLAS topography was carried out before surgery, on day 1 and weeks 1, 2, 3 and 4 following surgery. Comparison analysis tools of the device were used to calculate the change in refractive power, measured in diopters (D), in the center of the corneal inlay on day 1 and weeks 1, 2, 3 and 4 compared to before surgery. Three topography scans were carried out at each time point and the mean value of the 3 measurements calculated. The change in refractive power was compared between the 4 pocket and flocket groups, and also examined for change over time.

Astigmatism was measured in diopters on the axial curvature maps at the above time points. The average of the 3 scans was compared between the 4 corneal pocket/flocket groups and also examined for change over time.

#### In-vivo confocal microscopy

In vivo confocal microscopy (IVCM) was performed with the corneal module of the HRT3 (Heidelberg Engineering GmbH, Heidelberg, Germany) on the rabbit corneas before surgery and at weeks 1, 2, 3 and 4 after surgery. A carbomer gel (Vidisic; Mann Pharma, Berlin, Germany) was applied on the confocal lens and used as the immersion fluid.

Each cornea was examined in 3 locations: above, through and below the inlay, at the inlay edge and adjacent to the inlay (approximately 100 μm from the inlay edge). A minimum of three z-axis scans was carried out throughout the entire corneal thickness. For each cornea, 3 micrographs from each of the following areas were selected for analysis and calculation of mean reflectivity: at the inlay edge, adjacent to the inlay edge, 10 μm anterior to and 10 μm posterior to the area adjacent to the inlay. These scans were analyzed by semi-quantifying the mean gray value of reflectivity using Image J (http://imagej.nih.gov/ij/; provided in the public domain by the National Institutes of Health, Bethesda, MD, USA);[13,22] the mean value was then calculated and normalized to the mean value of the controls.

#### Immunohistochemistry and histology

After euthanasia at week 4, the rabbit corneas were excised, and one half was embedded in an optimal cutting temperature compound (Leica Microsystems, Nussloch, Germany) and stored at −80°C. Serial transverse corneal sections (8 μm thick) were cut with a cryostat (Microm HM550; Microm, Walldorf, Germany) and placed on polylysine-coated glass slides. The slides were stored at −80°C until immunofluorescence staining.

After thawing at room temperature, the corneal sections were air-dried. They were fixed with 4% neutral buffered paraformaldehyde (Sigma-Aldrich, St. Louis, MO, USA), washed with 1× PBS (first BASE, Singapore) and incubated in 1× PBS containing 0.15% Triton X-100 (Sigma-Aldrich) to increase cellular permeability. They were then incubated in 4% bovine serum albumin (Sigma-Aldrich) and incubated overnight at 4°C with primary antibodies. The antibodies were mouse monoclonal antibody against cellular fibronectin (5 μg/mL; Millipore Corp., Billerica, MA, USA), rat monoclonal antibody against CD11b (20 μg/ml; BD Pharmingen, Franklin Lakes, NJ, USA) and mouse monoclonal antibody against heat shock protein 47 (HSP47) (1 μg/ml Enzo Life Sciences, Switzerland). The following day, the slides were washed with 1× PBS and the sections incubated with either a goat anti-mouse or goat anti-rat AlexaFluor 488-conjugated secondary antibody (Life Technologies, Carlsbad, CA, USA) for 1 hour in room temperature. The slides were then washed with 1× PBS and mounted with UltraCruz Mounting Medium containing DAPI (Santa Cruz Biotechnology, Dallas, TX, USA). The sections were visualized and imaged using a fluorescence microscope (AxioImager Z1; Carl Zeiss, Oberkochen, Germany).

Corneal sections were also stained with Hematoxylin (Sigma-Aldrich) and Eosin (Sigma-Aldrich). Microscopic qualitative assessment was carried out under a light microscope (100X magnification).

#### Transmission electron microscopy

Corneal tissue measuring 1.5 x 1.5 mm was excised from the second half of the cornea and fixed overnight in 2% glutaraldehyde (Electron Microscopy Sciences, Hatfield, PA) at 4°C in preparation for transmission electron microscopy (TEM). The tissue was washed in sodium cacodylate buffer (Electron Microscopy Sciences, Hatfield, PA) for 10 minutes and rinsed copiously with distilled water. Post-fixation was then carried out in 1% osmium tetroxide (Electron Microscopy Sciences, Hatfield, PA) for 2 hours at room temperature. After rinsing with distilled water, the tissue was dehydrated in an increasing concentration of ethanol (from 25% to 50%, 75%, 95%, and 100%) and embedded in Araldite (Electron Microscopy Sciences, Hatfield, PA). Ultra-thin sections of 60 to 80 nm thickness were collected on copper grids, doubled-stained with uranylacetate and lead citrate for 10 minutes each, then viewed and photographed using a Philips EM 208S Transmission Electron Microscope (FEI Electron Optics BV, Eindhoven, the Netherlands).

#### Statistical analysis

Data were expressed as mean ± standard deviation (SD). Statistical comparisons among different groups were performed using Friedman two-way analysis of variance, Kruskal–Wallis and Wilcoxon sign rank tests. The Statistical Package for Social Science (IBM SPSS Statistics for Macintosh, Version 22.0. Armonk, NY: IBM Corp) was used. Statistical significance was considered p<0.05. When the Bonferroni correction was used for multiple comparisons, p<0.0083 was considered significant.

## Results

### Slit lamp biomicroscopy photography

All eyes showed no evidence of conjunctival injection after surgery. No corneal infiltration to suggest the development of corneal infection was detected. The corneal contour also appeared smooth and regular with no focal thinning to suggest stromal necrosis. The corneas of all rabbits remained clear with no stromal haze development.

### Anterior segment optical coherence tomography

**Fig 3:**
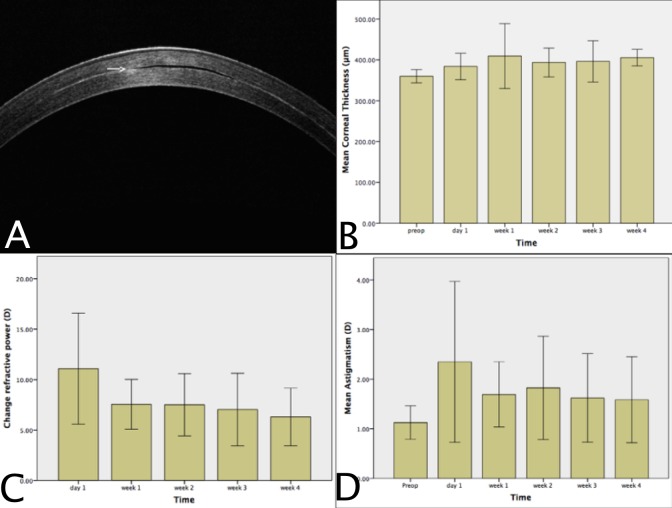
Corneal thickness, refractive power change and astigmatism following Raindrop inlay implantation. A. The optically transparent inlay (arrow) is non-reflective, resulting in the appearance of an empty stromal space. B. A significant increase in the mean corneal thickness of all eyes was observed by the first postoperative day compared to before surgery. Mean corneal thickness remained increased at all time points compared to before surgery and did not change significantly after day 1. C. The mean increase in refractive power of all eyes was significant as early as on the first postoperative day and remained significant at all time points; no significant change was observed between day 1 and week 1, weeks 1 and 2, weeks 2 and 3, and weeks 3 and 4. D. The mean astigmatism of all eyes was significantly increased at day 1 following surgery, but not at week 1 or later. (error bars: ± 1SD)

Central CT (mean ± SD) increased from 360.0 ± 16.2 μm before implantation to 383.9 ± 32.5, 409.4 ± 79.3, 393.6 ± 35.2, 396.4 ± 50.7 and 405 ± 20.3 μm on day 1 and weeks 1, 2, 3 and 4 respectively (p = 0.005), as illustrated in Fig 3. At all time points the increase was statistically significant compared to before surgery (p<0.008). There was no significant difference in central CT between day 1 and week 1, weeks 1 and 2, weeks 2 and 3, and weeks 3 and 4.

There was no significant difference in CT between the 4mm, 6mm, 8mm pocket and 8mm flocket groups at day 1 (397.8±29.6 vs. 351.5± 25.6 vs. 390.0±13.1 vs. 395.2±35.9, p = 0.136), week 1 (424.8±66.1 vs. 345.8±14.9 vs. 393.7±18.0 vs. 457.4±112.4, p = 0.081), week 2 (421.5±30.0 vs. 384.8±21.3 vs. 369.7±34.5 vs. 392.6±41.7, p = 0.270), week 3 (444.5±82.3 vs. 372.8±21.0 vs. 374.3±25.4 vs. 390.2±24.7, p = 0.678) and week 4 (412±22.2 vs. 398.3±12.6 vs. 396.7±27.2 vs. 412.5±22.2 p = 0.559).

### Anterior corneal surface topography

Corneal refractive power increased significantly from 50.3±3.8 D before implantation by 11.1±5.5, 7.5±2.5, 7.5±3.1, 7.0±3.6 and 6.3±2.9 D at day 1 and weeks 1, 2, 3 and 4 respectively following inlay implantation (p<0.001), as illustrated in Fig 3. The increase was significant (p<0.001) at all time points compared to before surgery. In multiple comparisons, the change was not statistically significant between day 1 and week 1 (p = 0.019), weeks 1 and 2 (p = 0.845), weeks 2 and 3 (p = 0.778), and weeks 3 and 4 (p = 0.171).

**Fig 4:**
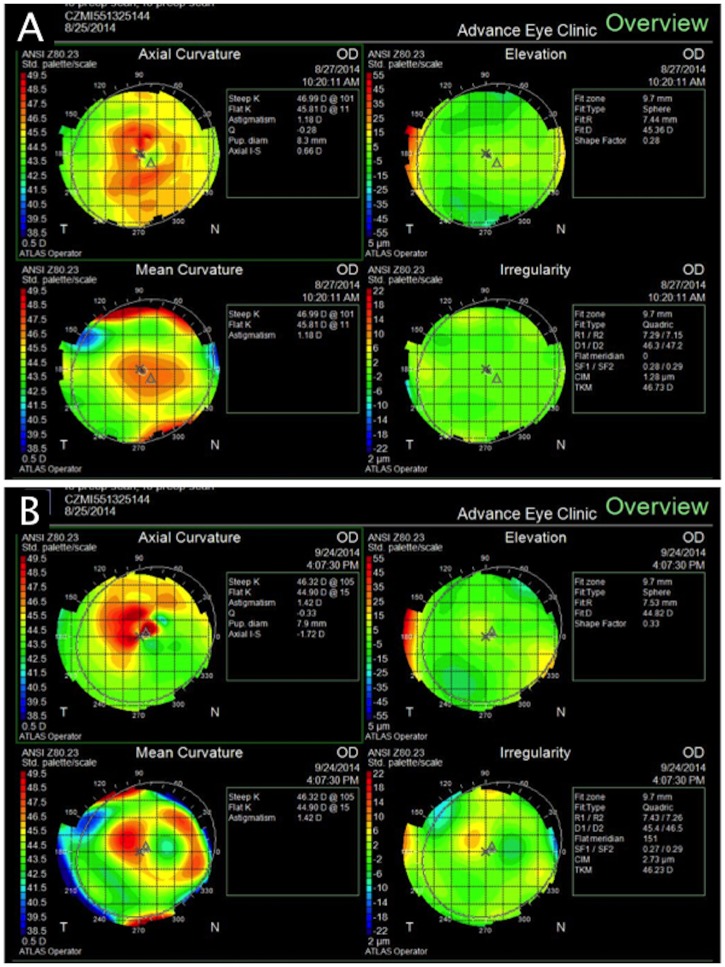
**Anterior corneal surface topography before (A) and 4 weeks (B) after Raindrop implantation in a 6mm pocket.** A focal increase in corneal curvature and irregularity that correspond to the position of the inlay were observed after implantation. A small increase in anterior elevation was also present.

There was a significant difference in the refractive power change between the 4mm, 6mm, 8mm pocket and 8mm flocket groups on day 1 (16.5±4.0 vs. 9.4±5.4 vs. 7.1±2.7 vs. 11.4±5.6, p = 0.049); multiple comparisons showed a significant difference only between the 4mm and 8mm pocket groups (p = 0.008). There was no significant difference in the refractive power change between the four groups at weeks 1 (8.7±1.6 vs. 6.0±3.2 vs. 7.1±2.2 vs. 8.5±2.3, p = 0.329), 2 (8.5±3.2 vs. 5.6±5.4 vs. 8.1±1.0 vs. 7.4±2.1, p = 0.519), 3 (8.4±3.4 vs. 4.3±0.9 vs. 8.8±5.2 vs. 6.7±2.5, p = 0.091) and 4 (7.3±2.8 vs. 6.3±5.4 vs. 5.0±1.2 vs. 6.5±1.2, p = 0.388). Anterior corneal surface topography before and after inlay implantation is illustrated in Fig 4.

Corneal astigmatism increased from 1.1±0.3 D before implantation to 2.3±1.6, 1.7±0.7, 1.8±1.0, 1.6±0.9 and 1.6±0.9D on day 1 and weeks 1, 2, 3 and 4 respectively (p = 0.033) (Fig 3). The increase in astigmatism was statistically significant only at day 1 (p = 0.006) and no other time point. There was no significant difference in astigmatism between the 4mm, 6mm, 8mm pocket and 8mm flocket groups at day 1 (2.0±1.4 vs. 3.3±2.4 vs. 1.8±1.2 vs. 2.1±1.2, p = 0.700), and weeks 1 (1.3±0.3 vs. 2.3±0.8 vs. 1.4±0.5 vs. 1.7±0.5, p = 0.086), 2 (1.2±0.4 vs. 1.6±0.8 vs. 1.7±0.3 vs. 2.8±1.6, p = 0.228), 3 (1.0±0.5 vs. 1.6±0.8 vs. 1.9±1.4 vs. 2.0±0.3, p = 0.131) and 4 (0.9±0.5 vs. 1.5±0.4 vs. 1.8±0.5 vs. 2.1±1.3, p = 0.064).

### In vivo confocal microscopy

**Fig 5:**
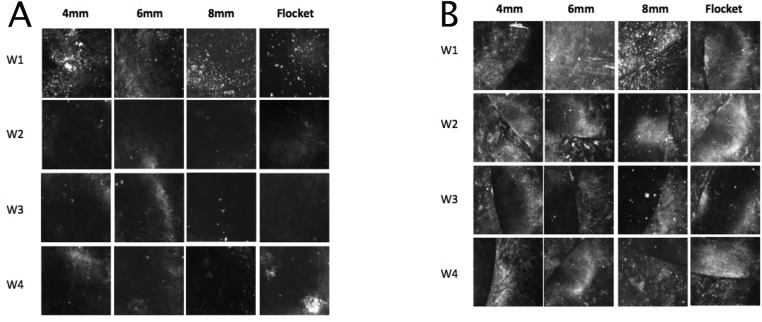
**In vivo confocal microscopy images at the inlay plane (A) and inlay edge (B).** Highly reflective particles were observed in all eyes and, although they were more apparent in the early stages post-implantation, they decreased with time. (W1 = week 1, W2 = week 2, W3 = week 3, W4 = week 4)

**Fig 6:**
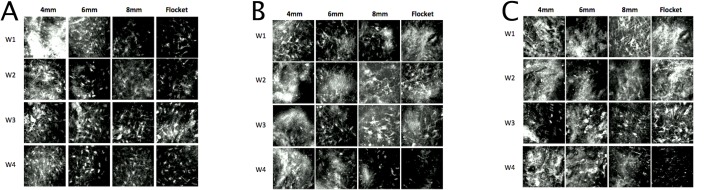
**Representative in vivo confocal microscopy micrographs for different time points at the areas anterior to the inlay edge (A), anterior to the inlay plane (B), and adjacent to the inlay (C).** Highly reflective keratocytes were seen in these areas surrounding the inlays. (W1 = week 1, W2 = week 2, W3 = week 3, W4 = week 4)

**Fig 7:**
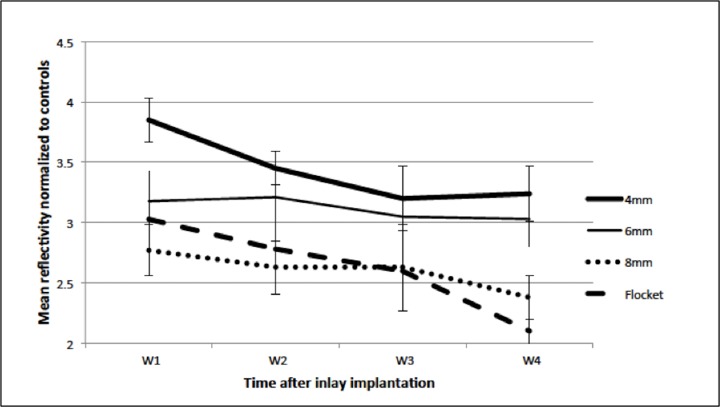
Stromal keratocyte mean reflectivity for the different pocket/flocket groups. The 4mm pocket group had significantly higher stromal keratocyte reflectivity than the other groups throughout the study period, whereas the 8mm pocket and flocket groups had lower reflectivity. (W1 = week 1, W2 = week 2, W3 = week 3, W4 = week 4)

The inlay plane was acellular and the inlay itself hypo-reflective. Highly reflective particles were observed at the inlay plane and inlay edge in all eyes, especially in the early stages post-implantation (Fig 5A and 5B). Highly reflective keratocytes were seen in the area adjacent to the inlays (Fig 6A, 6B and 6C). Semi-quantitative analysis of the reflectivity intensity of micrographs at the inlay edge, adjacent to the inlay edge, 10 μm anterior to and 10 μm posterior to the area adjacent to the inlay, as detailed above, showed that the 4mm pocket group had the highest mean stromal keratocyte reflectivity throughout the study period (p = 0.033, p<0.005, and p<0.005, compared to the 6mm, 8mm and flocket groups, respectively, at 4 weeks post-implantation) (Fig 7). At 4 weeks, the 6mm pocket group also had significantly higher mean stromal keratocyte reflectivity than the 8mm pocket and flocket groups (both p<0.005). Throughout the 4 weeks, the 8mm pocket and flocket groups had the least keratocyte reflectivity; there were no significant differences between the 8mm pocket and flocket groups at any time point. For all eyes, the mean keratocyte reflectivity gradually decreased with time (Fig 7).

#### Immunohistochemistry and histology

**Fig 8:**
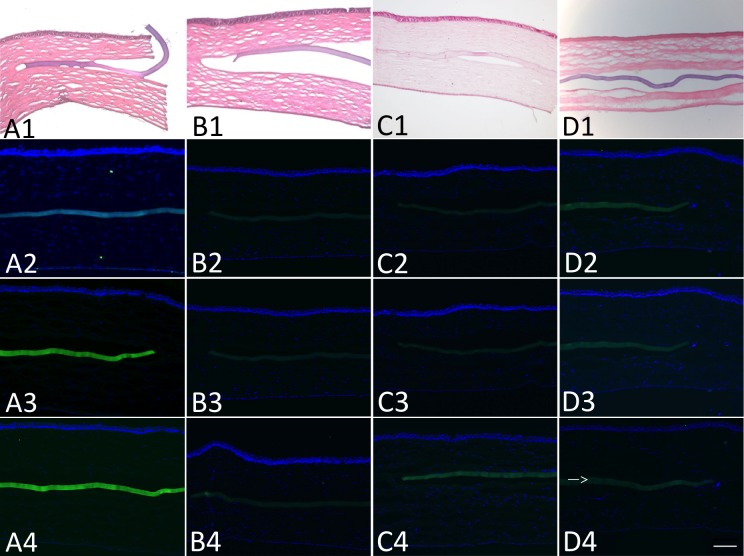
Hematoxylin and eosin stained corneal sections and expression of CD11b, fibronectin and HSP47 four weeks post-implantation. Hematoxylin and eosin stained sections of the 4mm pocket (A1), 6mm pocket (B1), 8mm pocket (C1) and 8mm flocket (D1) groups showed no inflammatory cells or fibrotic capsule formation around the inlay. No CD11b, fibronectin or HSP 47 positive cells were detected in the 4mm pocket (A2-4), 6mm pocket (B2-4J), 8mm pocket (C2-4) and 8mm flocket (D2-4) groups, respectively. Scale bar 100 μm. (arrow points to the inlay) Row 1 represents hematoxylin and eosin, row 2 CD11b, row 3 fibronectin and row 4 HSP47 corneal sections.

Microscopy of tissue sections of corneas that were excised at 4 weeks and stained with hematoxylin and eosin showed that the inlay was well placed in the stromal space and there were no inflammatory cells or fibrotic capsule present around the inlay (Fig 8). At 4 weeks post-implantation, there was no expression of CD11b, fibronectin or HSP47 in the implanted corneas (Fig 8), indicating that the inlay elicited no detectable inflammatory reaction, fibrotic response or tissue stress response.

### Transmission electron microscopy

**Fig 9:**
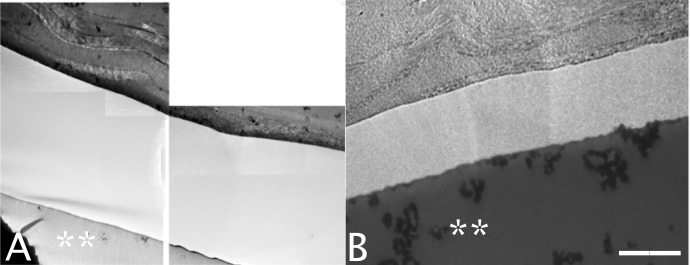
**Representative Transmission Electron Microscopy micrographs of the collagen fibers in the 6mm pocket (A) and flocket groups (B).** The collagen fibers of the corneal stroma surrounding the inlay were intact and regular with no disruption or distortion in all groups. Scale bar 5 μm. (asterisk indicates inlay)

The TEM micrographs showed the ultrastructure of the collagen fibers surrounding the inlay. In all four groups, the collagen fibers were intact and had a regular arrangement, without being distorted or disrupted by the implanted inlay (Fig 9).

## Discussion

Our study of Raindrop™ implantation in a range of corneal pockets showed that the cornea achieved anatomical and refractive stability by 1 week following surgery. Implantation of the inlay in a pocket or flocket did not affect the anatomical and refractive outcomes, nor did pocket size. Although we did not find an inflammatory or scarring response with either laser platform, we did detect keratocyte activation that was least in the 8mm pocket and flocket groups.

The central CT increased on week 1 following implantation and did not change significantly thereafter. The pocket and flocket dimensions did not affect the postoperative CT. This increase in CT developed most likely due to the space occupying effect of the inlay, as it was consistent with the central inlay thickness of 32 μm. The Raindrop™, a space-occupying and refractively neutral inlay, improves near vision by increasing the curvature of the anterior corneal surface, creating a hyper-prolate region of increased refractive power.[3] The increase in refractive power, observed as early as 1 day following surgery, also stabilized by the first week. This was similar to a clinical report with implantation of the Raindrop™ inlay under a flap, where an improvement in uncorrected near vision as early as 1 day postoperatively and stabilization at 1 week were found.[19]

We found that the dimension and configuration of the pocket or flocket did not affect the inlay-induced increase in refractive power. This suggests that a variety of pocket or flocket configurations and femtosecond laser platforms may potentially be used with the Raindrop™. In our rabbit animal study, a refractive power increase of 6.3 D was present at the fourth postoperative week. This is greater than the effect of inlay implantation in the human eye, where typically a maximum near add effect of 3 D is expected, although wavefront aberrometry has measured a refractive effect of approximately 4.5 D in the center of the pupil with the Raindrop™.[18] The elastic properties of the rabbit cornea, and the very thin Bowman’s layer,[23] may allow for a greater increase in the anterior corneal curvature, and thus refractive power, to be effected. Our findings, therefore, need to be interpreted in the context of the animal nature of the study. Since our objective was to compare the effects of different pocket configurations, this was achievable in a controlled animal study and would be difficult to achieve in a controlled clinical trial.

Astigmatism increased temporarily on the first day, but there was no significant increase at week 1 or later. The pocket/flocket configuration did not affect astigmatism. Previous clinical studies have placed the Raindrop™ inlay under a corneal flap greater than 8 mm in diameter.[18–21] The use of femtosecond laser technology has enabled clinicians to place presbyopic inlays in pockets with a diameter as small as 3.6 and 4.2 mm;[14,24] 4 mm was the smallest pocket that could be achieved with the Ziemer FEMTO LDV Z6. Implantation of the Raindrop™ in a pocket has not been reported. The use of a pocket rather than a flap may potentially be associated with less postoperative dry eye. It is well documented that small incision lenticule extraction (SMILE) for refractive correction, which does not involve a flap, results in less dry eye and better preservation of the corneal nerves and sensation than LASIK.[15–17] We would hence expect the same effect with the use of corneal pockets for Raindrop™ implantation rather than with flaps.

The Raindrop™ inlay did not induce corneal inflammation or scarring in any group. All 4 surgical groups received the same post-operative steroid regimen, 1% prednisolone acetate drops for 4 weeks, in order to investigate the effect of different pockets/flocket dimensions. In clinical studies, the duration of post-operative steroid use has ranged from 1 week to 3 months.[18–20] Immunohistochemistry did not detect CD11b, fibronectin or HSP47, markers of granulocyte leukocytes, corneal fibrosis,[25] and active collagen synthesis,[26] respectively. A rabbit study that investigated the Kamra™ inlay found no inflammation at 6 weeks following implantation.[27] Our pocket results are similar to the clinical results following flap implantation. In a study with the Raindrop™ inlay, involving 20 eyes, there were no cases of clinical corneal inflammation or scarring.[18] However, when implantation was combined with either hyperopic LASIK,[19] or myopic LASIK,[21] clinical haze was found in 1 of 13 hyperopic treated eyes and 1 of 30 myopic treated eye. This may be circumvented in the future by combining a pocket insertion with conventional hyperopic or myopic LASIK.

We found that keratocyte activation decreased rapidly from week 1 to 4, but remained slightly elevated compared to untreated controls at week 4. Activation was least in the 8 mm pocket and flocket groups, indicating that the early increased keratocyte activation is likely from the surgical manipulation involved with inlay implantation through the longer access tunnels of the tighter 4 mm and 6 mm pockets. Sham manual pocket creation without an implant has previously been found to been associated with mild keratocyte activation.[28] We did not include a flap group in our study, as implantation under a flap is already in clinical use and our aim was to investigate a range of novel pocket/flocket configurations with minimal use of animals (3Rs principle).

All our pockets were created with a nJ femtosecond laser and we have previously shown minimal keratocyte activation following lamellar creation with this laser.[13] It is very unlikely that the keratocyte activation observed in our study was related to inlay induced inflammation, as no inflammatory cells were identified with immunohistochemistry and histology to suggest a foreign body reaction. We did not specifically examine for transdifferentiation of stromal keratocytes, but fibronectin that is produced by myofibroblasts was not detected. The in vivo confocal microscopy supports good short-term biocompatibility for the Raindrop™ inlay. The literature, however, suggests that inlays may induce a subtle but continuous activation response in stromal keratocytes. Mild keratocyte activation has been detected up to 12 months following Flexivue Microlens™ implantation.[29] Limnopoulou et al, however, showed no keratocyte activation also 12 months following Flexivue Microlens™ implantation.[24] Our study did not examine such long-term effects.

In conclusion, this is the first study to examine the wound and refractive response following implantation of the Raindrop™ inlay in a range of corneal pockets. The Raindrop™ inlay produced an increase in corneal refractive power that was not affected by the dimension and configuration of the pocket or flocket. Keratocyte activation developed post-implantation but this decreased to mild levels by 4 weeks; no inflammatory response was elicited. The least keratocyte activation was observed in the 8mm pocket and flocket groups, suggesting that 8mm may be the preferred dimension; clinical studies are required to verify this.

## References

[1] Ostrin LA, Glasser A. Accommodation measurements in a prepresbyopic and presbyopic population. J Cataract Refract Surg 2004; 30:1435–44. 10.1016/j.jcrs.2003.12.045 15210220

[2] Holden BA, Fricke TR, Ho SM, Wong R, Schlenther G, Cronjé S, et al Global vision impairment due to uncorrected presbyopia. Arch Ophthalmol 2008; 126:1731–9. 10.1001/archopht.126.12.1731 19064856

[3] Konstantopoulos A, Mehta JS. Surgical compensation of presbyopia with corneal inlays. Expert Rev Med Devices. 2015;12:341–52. 10.1586/17434440.2015.1007124 25652889

[4] Ito M, Shimizu K, Amano R, Handa T. Assessment of visual performance in pseudophakic monovision. J Cataract Refract Surg 2009;35:710–4. 10.1016/j.jcrs.2008.12.019 19304093

[5] Finkelman YM, Ng JQ, Barrett GD. Patient satisfaction and visual function after pseudophakic monovision. J Cataract Refract Surg 2009;35:998–1002. 10.1016/j.jcrs.2009.01.035 19465283

[6] Wilkins MR, Allan BD, Rubin GS, Findl O, Hollick EJ, Bunce C, et al; Moorfields IOL Study Group. Randomized trial of multifocal intraocular lenses versus monovision after bilateral cataract surgery. Ophthalmology 2013;120:2449–2455. 10.1016/j.ophtha.2013.07.048 24070808

[7] van der Linden JW, van Velthoven M, van der Meulen I, Nieuwendaal C, Mourits M, Lapid-Gortzak R. Comparison of a new-generation sectorial addition multifocal intraocular lens and a diffractive apodized multifocal intraocular lens. J Cataract Refract Surg 2012;38:68–73. 10.1016/j.jcrs.2011.06.034 22078117

[8] van der Linden JW, van der Meulen IJ, Mourits MP, Lapid-Gortzak R. Comparison of a hydrophilic and a hydrophobic apodized diffractive multifocal intraocular lens. Int Ophthalmol 2013;33:493–500. 10.1007/s10792-013-9727-5 23381387 PMC3782640

[9] Ryan A, O'Keefe M. Corneal approach to hyperopic presbyopia treatment: six-month outcomes of a new multifocal excimer laser in situ keratomileusis procedure. J Cataract Refract Surg. 2013;39:1226–33. 10.1016/j.jcrs.2013.03.016 23747205

[10] Menassa N, Fitting A, Auffarth GU, Holzer MP. Visual outcomes and corneal changes after intrastromal femtosecond laser correction of presbyopia. J Cataract Refract Surg. 2012;38:765–773. 10.1016/j.jcrs.2011.11.051 22520302

[11] Luger MH, Ewering T, Arba-Mosquera. One-year experience in presbyopia correction with biaspheric multifocal central presbyopia laser in situ keratomileusis. Cornea. 2013;32:644–52. 10.1097/ICO.0b013e31825f02f5 23086358

[12] Chen S, Feng Y, Stojanovic A, Jankov MR 2nd, Wang Q. IntraLase femtosecond laser vs mechanical microkeratomes in LASIK for myopia: a systematic review and meta-analysis. J Refract Surg. 2012;28:15–24. 10.3928/1081597X-20111228-02 22233436

[13] Riau AK, Liu YC, Lwin NC, Ang HP, Tan NY, Yam GH, et al Comparative study of nJ- and μJ-energy level femtosecond lasers: evaluation of flap adhesion strength, stromal bed quality, and tissue responses. Invest Ophthalmol Vis Sci. 2014;55:3186–3194. 10.1167/iovs.14-14434 24764066

[14] Baily C, Kohnen T, O'Keefe M. Preloaded refractive-addition corneal inlay to compensate for presbyopia implanted using a femtosecond laser: one-year visual outcomes and safety. J Cataract Refract Surg. 2014;40:1341–8. 10.1016/j.jcrs.2013.11.047 25088635

[15] Denoyer A, Landman E, Trinh L, Faure JF, Auclin F, Baudouin C. Dry eye disease after refractive surgery: comparative outcomes of small incision lenticule extraction versus LASIK. Ophthalmology. 2015;122:669–76. 10.1016/j.ophtha.2014.10.004 25458707

[16] Li M, Zhao J, Shen Y, Li T, He L, Xu H, et al Comparison of dry eye and corneal sensitivity between small incision lenticule extraction and femtosecond LASIK for myopia. PLoS One. 2013;8(10):e77797 10.1371/journal.pone.0077797 24204971 PMC3812215

[17] Mohamed-Noriega K, Riau AK, Lwin NC, Chaurasia SS, Tan DT, Mehta JS. Early corneal nerve damage and recovery following small incision lenticule extraction (SMILE) and laser in situ keratomileusis (LASIK). Invest Ophthalmol Vis Sci. 2014;55:1823–34. 10.1167/iovs.13-13324 24569584

[18] Garza EB, Gomez S, Chayet A, Dishler J. One-year safety and efficacy results of a hydrogel inlay to improve near vision in patients with emmetropic presbyopia. J Refract Surg. 2013;29:166–72. 10.3928/1081597X-20130129-01 23446012

[19] Chayet A, Barragan Garza E. Combined hydrogel inlay and laser in situ keratomileusis to compensate for presbyopia in hyperopic patients: one-year safety and efficacy. J Cataract Refract Surg. 2013;39:1713–21. 10.1016/j.jcrs.2013.05.038 24021565

[20] Yoo A, Kim JY, Kim MJ, Tchah H. Hydrogel Inlay for Presbyopia: Objective and Subjective Visual Outcomes. J Refract Surg. 2015;31:454–60. 10.3928/1081597X-20150623-03 26158925

[21] Garza EB, Chayet A. Safety and efficacy of a hydrogel inlay with laser in situ keratomileusis to improve vision in myopic presbyopic patients: one-year results. J Cataract Refract Surg. 2015;41:306–12. 10.1016/j.jcrs.2014.05.046 25661123

[22] Liu YC, Jayawinghe L, Ang HP, Lwin NC, Yam GH, Mehta JS. Effect of intraoperative corneal stromal pocket irrigation in small incision lenticule extraction. BioMed Res Int 2015; 928608. 10.1155/2015/928608 PMC4530243 26273659

[23] Hayashi S, Osawa T, Tohyama K. Comparative observations on corneas, with special reference to Bowman's layer and Descemet's membrane in mammals and amphibians. J Morphol. 2002;254:247–58. 10.1002/jmor.10030 12386895

[24] Limnopoulou AN, Bouzoukis DI, Kymionis GD, Panagopoulou SI, Plainis S, Pallikaris AI, et al Visual outcomes and safety of a refractive corneal inlay for presbyopia using femtosecond laser. J Refract Surg. 2013;29:12–8. 10.3928/1081597X-20121210-01 23311737

[25] Mohan RR, Tandon A, Sharma A, Cowden JW, Tovey JC. Significant inhibition of corneal scarring in vivo with tissue-selective, targeted AAV5 decorin gene therapy. Invest Ophthalmol Vis Sci. 2011;52:4833–41. 10.1167/iovs.11-7357 21551414 PMC3175954

[26] Iwano M, Plieth D, Danoff TM, Xue C, Okada H, Neilson EG. Evidence that fibroblasts derive from epithelium during tissue fibrosis. J Clin Invest. 2002;110:341–50. 10.1172/JCI15518 12163453 PMC151091

[27] Santhiago MR, Barbosa FL, Agrawal V, Binder PS, Christie B, Wilson SE. Short-term cell death and inflammation after intracorneal inlay implantation in rabbits. J Refract Surg. 2012;28:144–9. 10.3928/1081597X-20111122-02 22149664

[28] Tan XW, Hartman L, Tan KP, Poh R, Myung D, Zheng LL, et al In vivo biocompatibility of two PEG/PAA interpenetrating polymer networks as corneal inlays following deep stromal pocket implantation. J Mater Sci Mater Med. 2013;24:967–77. 10.1007/s10856-012-4848-3 23354737 PMC3620449

[29] Malandrini A, Martone G, Canovetti A, Menabuoni L, Balestrazzi A, Fantozzi C, et al Morphologic study of the cornea by in vivo confocal microscopy and optical coherence tomography after bifocal refractive corneal inlay implantation. J Cataract Refract Surg. 2014;40:545–57. 10.1016/j.jcrs.2013.08.057 24680518

